# Surgical Outcomes of Low Anterior Resection Using the Senhance Surgical System: A Single‐Center Case Series

**DOI:** 10.1111/ases.70221

**Published:** 2025-12-22

**Authors:** Takatsugu Fujii, Yasumitsu Hirano, Yasuhiro Ishiyama, Sohei Akuta, Yusuke Nishi, Akihito Nakanishi, Hisashi Hayashi, Yume Minagawa, Hirofumi Sugita, Chikashi Hiranuma

**Affiliations:** ^1^ Department of Gastrointestinal Surgery Saitama Medical University International Medical Center Hidaka Saitama Japan

**Keywords:** laparoscopic surgery, rectal cancer, robotic surgery, Senhance surgical system

## Abstract

**Introduction:**

The Senhance Surgical System is the second robotic surgical platform approved for laparoscopic procedures in Japan. It is unique compared to other systems, offering eye‐tracking camera control, haptic feedback, and reusable instruments. Although its application in colon surgery has shown favorable outcomes, reports on its utility in rectal cancer surgery are limited. We aimed to evaluate the surgical outcomes of patients who underwent Senhance‐assisted low anterior resection for rectal cancer.

**Materials and Surgical Technique:**

We retrospectively analyzed 16 patients who underwent low anterior resection for rectal cancer using the Senhance system between February 2020 and December 2022. Surgical indications included resectable rectal cancer without adjacent organ invasion or emergency presentation. Demographic data, perioperative outcomes, pathological findings, and recurrence were assessed. The median duration of the operation was 308.5 min, with minimal blood loss (median 0 mL). Conversion to laparoscopy occurred in 5 patients (31.3%) due to pelvic dissection difficulty. There was no case of conversion to open surgery. No Clavien–Dindo grade ≥ 2 complications were observed. The median duration of hospitalization was 8 (6–14) days. R0 resection was achieved in 14 cases, and R2 resection was performed in 2 cases with synchronous distant metastases. The median number of harvested lymph nodes was 22. Four patients (28.6%) experienced recurrence, excluding those with stage IV disease.

**Discussion:**

Senhance‐assisted low anterior resection can be safely performed with acceptable short‐term oncological outcomes. However, its limitations in deep pelvic dissection and system setup require further investigation. Larger studies are needed to validate its utility in rectal surgery.

## 1 Introduction

Laparoscopic surgery is now widely recognized as a standard treatment for rectal cancer, as it can promote faster postoperative recovery and help maintain patients' quality of life [1]. However, pelvic dissection remains technically demanding because of instrument rigidity, restricted visualization, and ergonomic burden, especially in patients with a narrow pelvis.

The Senhance surgical system (Asensus Surgical, Durham, NC, USA) is the second robot‐assisted platform covered by the National Health Insurance in Japan. Its unique features include eye‐tracking camera control, haptic feedback, independent movable arms, and a laparoscopic‐like interface [2, 3]. Additionally, its reusable instruments and lower maintenance costs offer economic advantages over other robotic platforms.

Since its introduction, Senhance has shown feasibility and safety in gastrointestinal surgery [4, 5], with increasing applications in colorectal cancer. According to our earlier work, this system achieved promising short‐ to mid‐term results in colon surgery [2, 5]. However, studies on its use in rectal cancer are limited [6, 7, 8].

This retrospective study aimed to determine the surgical outcomes of patients who underwent Senhance‐assisted low anterior resection (LAR) for rectal cancer, and to assess the safety, feasibility, and technical limitations of the system when used in rectal cancer surgery.

## 2 Materials and Surgical Technique

Between February 2020 and December 2022, 16 patients underwent Senhance‐assisted LAR for rectal cancer at a single medical center. The inclusion criterion was resectable rectal cancer treated using LAR as a planned approach. Patients with invasion of adjacent organs and those who underwent emergency surgery were excluded from the analysis. Patient demographics, preoperative treatments, staging, operative details, complications, pathology, and recurrence were retrospectively reviewed.

## 3 Surgical Technique

A 3‐cm incision was made at the umbilical site.

A multichannel port for the camera and assistant instruments was inserted through this incision.

**FIGURE 1:**
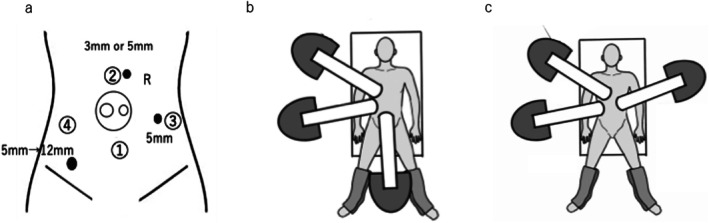
(a–c) Senhance digital laparoscopy system. (a) ① Port configuration for Senhance‐assisted low anterior resection. A multichannel umbilical port and additional 3‐to 5‐mm ports were arranged as shown. Figure ②–④: Port configuration for Senhance‐assisted rectal surgery. A 5‐mm port in the right lower quadrant and a 3‐mm port in the left lower quadrant. (b) Three robotic arms are placed on the patient's right side. The patient's height is adjusted during docking to maximize the range of motion of the robotic arms. (c) At the transition to pelvic dissection, two robotic arms are positioned on the patient's right side and one arm on the left side.

Two additional 5‐mm ports were inserted: one in the right lower quadrant and the other in the epigastric region (Figure 1a).

The Patient‐side Surgeon is responsible for auxiliary manipulation of forceps and instrument exchange. Importantly, because the three robotic arms have different ranges of motion, it is essential to adjust them according to the operative field to maximize their mobility.

Medial dissection toward the inferior mesenteric artery (IMA) was performed first, assisted by an organ retractor or forceps (Figure 1b). High ligation of the inferior mesenteric artery (IMA) was performed using an advanced energy device, such as an advanced bipolar or ultrasonic device.

During pelvic dissection, the left robotic arm was repositioned to a port at the umbilical level on the left abdomen or to the left lower abdominal port, and the procedure was performed (Figure 1c). At this stage, rectal exposure was achieved by grasping and controlling the gauze wrapped around the rectum with forceps inserted through the epigastric port by the assistant. Rectal transection was performed by replacing the right lower abdominal port with a 12‐mm port, through which a stapler was introduced (File S1).

## 4 Results

The median age of the 16 patients (8 males and 8 females) was 65.5 years (range, 43–80) (Table 1). The median duration of the operation was 308.5 min. The median blood loss was 0 mL, which was negligible.

**TABLE 1:** Patient characteristics.

*n*		16
Age	Median (range)	65.5 (43–80)
Sex	Male	8 (50.0)
	Female	8 (50.0)
Anal verge	0–5 cm	1 (6.3)
	6–10 cm	8 (50.0)
	11–15 cm	7 (43.8)
BMI median (range)		20.7 (18.0–31.0)
ASA	Class 1/2/3	
cT	1/2/3/4	3/4/8/1
cN	1/2/3/4	8/7/0/1
Ileostomy		7 (43.8)
Adjuvant chemotherapy		3 (18.8)

Abbreviations: ASA, American Society of Anesthesiologists; BMI, body mass index.

Five patients (31.3%) required conversion to laparoscopy because of difficulties in pelvic dissection. Conversion to open surgery was not required. There were no Clavien–Dindo grade ≥ 2 complications. The median length of hospital stay was 8 days (range: 6–14). R0 resection was achieved in 14 cases, and R2 resection in two patients with synchronous distant metastases. No R1 resections were performed. Pathological examination revealed predominantly well‐ or moderately differentiated adenocarcinomas. No poorly differentiated tumors were observed.

The median number of harvested lymph nodes was 22 (range: 7–26). The final staging included stages I (*n* = 5), II (*n* = 4), III (*n* = 5), and IV (*n* = 2). The median tumor size was 4.0 cm (range, 0–7.0 cm). Adjuvant chemotherapy was administered in six high‐risk stage II and III cases. Excluding stage IV, four patients (28.6%) developed recurrence during a median follow‐up of 36 months (range: 36–48), including one case of anastomotic recurrence (Tables 2 and 3). In the case of anastomotic recurrence (Case 8), the tumor was located in the lower rectum, but pathological examination confirmed negative margins, and the distal margin was 2 cm.

**TABLE 2:** Surgical outcomes.

*n*		16
Histological type *n* (%)	tub1.2	15
	muc	1
pT	1/2/3/4	3/6/5/2
pN	0/1/2/3	12/0/3/1
f‐Stage	1/2/3/4	8/3/3/2
R0/1/2		14/0/2
Operative time (min)	Median (range)	308.5 (240–495)
Intraoperative hemorrhage (mL)	Median (range)	0 (0–100)
Lymph node harvested	Median (range)	22.0 (7–26)
Complications [*n* (%)]	Clavien‐Dindo grade ≥ 2	0
Length of stay in days	Median (range)	8 (6–14)
	f‐Stage 1/2/3/4	1/3/2/2
Recurrence	Total	5
Recurrence site	Lung	2
	Liver	1
	Lymph node	1
	Anastomosis Recurrence	1

**TABLE 3:** Report of surgical outcomes in a case series.

No	Sex	Age	BMI	ASA	Anal verge	Tumor size	Conversion to laparoscopy	Operative time	Intraoperative hemorrhage (mL)	Ileostomy	pT	pN	M	fStage	Pistoperative stay (day)	Adjuvant chemotherapy	Recurrence	RFS (month)
1	F	54	24.9	1	5.0	2.0	+	344	0	+	2	0	0	1	9	−	−	44
2	F	60	18.0	1	7.0	3.9	+	397	0	+	2	0	0	1	14	−	−	42
3	F	78	19.5	1	8.0	2.8	+	290	5	+	2	0	0	1	9	−	+ (Lung)	48
4	F	73	18.5	1	9.0	3.0	−	252	0	−	2	0	0	1	7	−	−	33
5	F	58	19.0	1	10.0	4.0	−	268	5	−	2	0	0	1	7	−	−	33
6	F	65	19.2	1	8.0	3.0	−	240	0	−	2	1a	0	3a	7	+	+ (Liver)	14
7	M	77	19.7	2	11.0	4.1	−	250	5	−	3	0	0	2a	10	−	−	34
8	M	48	24.7	2	13.0	5.0	−	459	50	−	3	0	0	2a	12	−	+ (Anastomosis)	35
9	M	70	19.1	1	15.0	5.0	−	327	0	−	3	0	0	2a	7	−	−	36
10	M	66	31.0	2	15.0	7.0	+	283	12	−	3	2a	0	3b	7	+	+ (Lung)	33
11	M	61	22.3	2	15.0	7.0	−	250	0	−	3	3	0	3c	8	+	+ (Lympho node)	42
12	M	73	21.4	1	9.0	N/A	−	384	0	+	1b	0	0	1	8	−	−	33
13	M	61	23.6	2	15.0	2.0	−	337	5	−	1b	0	0	1	8	−	−	33
14	F	43	20.4	2	15.0	N/A	+	259	0	+	1b	0	0	1	10	−	−	40
15	M	78	20.9	3	8.0	5.0	−	358	100	−	4a	1b	1a (Liver)	4a	9	−	NA	17
16	F	80	21.6	2	8.0	4.0	−	390	50	+	4a	0	1a (Lung)	4a	8	−	NA	34

Abbreviation: N/A, no residual carcinoma.

## 5 Discussion

Although laparoscopic surgery has been established as a standard technique for rectal cancer treatment, deep pelvic dissection remains technically demanding, particularly in patients with a narrow pelvis, obesity, or severe adhesions. In contrast, robotic‐assisted surgery, such as the da Vinci system, has demonstrated advantages including reduced blood loss and lower conversion rates, and has been evaluated as a viable alternative [9, 10, 11].

The results of this study show that total mesorectal excision (TME) using the Senhance Surgical System is safe and feasible for low anterior resection in patients with resectable rectal cancer. However, some cases required conversion to conventional laparoscopy, suggesting possible limitations of the system depending on patient and anatomical factors.

The Senhance system, based on the principles of laparoscopic surgery, offers unique advantages such as eye‐tracking camera control, haptic feedback, and cost‐effectiveness through reusable instruments [12]. Nevertheless, the lack of articulated instruments limits multidirectional adjustment and wide exposure in the deep pelvis. In particular, interference between the instruments and the camera made it difficult to obtain an adequate surgical field and perform dissection along the anterior and lateral rectal walls. Moreover, the conversion cases included not only patients with low rectal tumors but also individuals with high BMI or adhesions due to endometriosis, suggesting that patient factors beyond pelvic depth may influence the complexity of pelvic dissection.

Although the introduction of a fourth robotic arm was considered, it was infeasible because of spatial constraints. Therefore, applying the Senhance system in lower rectal cancer requires careful case selection, optimization of port placement and surgical technique, and team proficiency. Further challenges include developing adjunctive instruments and refining instrument‐handling techniques to enhance exposure in the deep pelvis. A possible solution may be to combine transanal TME (TaTME) with the Senhance system, as previous reports have shown that this hybrid approach is technically feasible [13, 14].

Regarding colorectal surgery using the Senhance platform, McKechnie et al. [15] reported a median operative time of 229 min, blood loss of 37 mL, and a 4.0% conversion rate to open surgery, findings that largely align with our results. Similarly, Samalavicius et al. [6] reported safe completion of TME without conversion, while Spinelli et al. [8] demonstrated oncologically acceptable outcomes, including R0 resection and adequate lymph node harvest.

The present study had several limitations. It was conducted at a single institution with a retrospective design and a small sample size. Multiple surgeons were involved, and differences in surgeon and assistant experience may have affected the operative time. Additionally, differences in tumor location and size may have influenced surgical outcomes, such as operative duration and blood loss. Learning curves and long‐term outcomes were not evaluated, limiting the generalizability of the findings. Further validation through multicenter studies or comparative trials is warranted.

In conclusion, Senhance‐assisted low anterior resection can be safely performed with acceptable short‐term oncological outcomes. However, limitations remain regarding deep pelvic manipulation and system setup. In cases where pelvic dissection becomes challenging, assistant intervention or the incorporation of TaTME may be necessary. However, integrating TaTME may negate the cost advantage of the Senhance system achieved through reusable instruments. Based on the findings of this study, the use of Senhance in low anterior resection depends on case selection and surgeon experience. Based on our experience, the current Senhance system may be better suited for upper to mid rectal cancers, whereas lower rectal tumors requiring deep pelvic dissection remain technically challenging due to the system's structural limitations. Consequently, its widespread adoption as a standard technique in rectal cancer surgery still faces challenges.

## Author Contributions

Y.I., T.F., and Y.H. designed the study and conducted the experiments. Y.I., Y.H., and T.F. drafted the manuscript. T.F. prepared the original draft of the manuscript. Y.I. supervised the study. All authors approved the final manuscript.

## Funding

The authors have nothing to report.

## Ethics Statement

This study was approved by the Institutional Review Board of Saitama Medical University International Medical Center (approval no. 2021‐088).

## Consent

Written informed consent was obtained from all patients prior to enrollment, including consent for the publication of anonymized clinical information.

## Conflicts of Interest

Yasumitsu Hirano: Consultant (Self): (Asensus Surgical), Honoraria (Self): (Asensus Surgical). Takatsugu Fujii, Yasuhiro Ishiyama, Sohei Akuta, Yusuke Nishi, Akihito Nakanishi, Hisashi Hayashi, Yume Minagawa, Hirofumi Sugita, and Chikashi Hiranuma have no competing interests.

## Supporting information


**File S1:** Edited video of Senhance‐assisted low anterior resection (5 min, double speed).

## Data Availability

The data that support the findings of this study are available on request from the corresponding author. The data are not publicly available due to privacy or ethical restrictions.
